# Accelerated Oral Healing by *Angelica gigas* Nakai from Hot Melt Extrusion Technology: An In Vitro Study

**DOI:** 10.3390/medicina59122066

**Published:** 2023-11-23

**Authors:** Ju Ri Ye, Ha Yeon Lee, Yea-Jin Park, Yong Kwon Chae, Hyo-Jin An, Jong-Suep Baek, Ok Hyung Nam

**Affiliations:** 1 Department of Pediatric Dentistry, Kyung Hee University Medical Center, College of Dentistry, Kyung Hee University, Seoul 02447, Republic of Korea; jjuri0303@gmail.com (J.R.Y.); pedochae@gmail.com (Y.K.C.); 2 Department of Bio-Health Convergence, Kangwon National University, Chuncheon 24341, Republic of Korea; lhy101624@kangwon.ac.kr; 3 Department of Rehabilitative Medicine of Korean Medicine and Neuropsychiatry, College of Korean Medicine, Sangji University, Wonju 26339, Republic of Korea; wer0928@hanmail.net; 4 Department of Oriental Pharmaceutical Science, College of Pharmacy, Kyung Hee University, Seoul 02447, Republic of Korea; hjan@khu.ac.kr; 5 BeNatureBioLab, Chuncheon 24206, Republic of Korea; 6 Department of Pediatric Dentistry, School of Dentistry, Kyung Hee University, Seoul 02447, Republic of Korea

**Keywords:** wound healing, natural product, hot melt extrusion technology, biocompatible materials

## Abstract

*Background and Objectives*: In spite of the oral environment being healing-prone, its dynamic changes may affect wound healing. The purpose of this study was to assess the oral wound healing effect of *Angelica gigas* Nakai (AG) prepared by hot-melt extrusion. *Materials and Methods*: Human gingival fibroblast (HGF) cells were treated with AG or AG via hot-melt extrusion (AGH) for 24 h to determine the optimal concentration. For evaluating the anti-inflammatory effect of AG and AGH, a nitric oxide assay was performed under lipopolysaccharide (LPS) stimulation. The wound-healing effects of AG and AGH were evaluated using cell proliferation/migration assays and wound-healing marker expression through qRT-PCR. *Results*: Both AG and AGH showed no cytotoxicity on HGH cells. Regarding nitric oxide production, AGH significantly decreased LPS-induced nitric oxide production (*p* < 0.05). AGH showed a significantly positive result in the cell proliferation/cell migration assay compared with that in AG and the control. Regarding wound healing marker expression, AGH showed significantly greater VEGF and COL1α1 expression levels than those in the others (*p* < 0.05), whereas α-SMA expression was significantly different among the groups. *Conclusions*: Within the limits of this study, AGH accelerated oral wound healing in vitro.

## 1. Introduction

As the oral cavity is lined with mucosal tissue and oral saliva can release various growth hormones and cytokines, oral wound healing is usually rapid [1]. However, the oral cavity is a dynamic environment characterized by constant changes in temperature and oral flora throughout life [2]. Tissues moving during speaking, chewing, and swallowing are key features of the oral cavity that can potentially affect oral wound healing. Infection may occur and impede oral wound healing if the wounded oral tissues are persistently irritated by these features [3]. Moreover, oral wound healing materials are clinically necessary for the dental surgery [4,5]. For example, a free gingival graft is a dental procedure used to induce intraoral soft tissue augmentation when the periodontal support is compromised [6]. The palate is frequently indicated as a donor site for free gingival grafts. The duration of palatal wound healing is reported to be approximately 2–4 weeks after free gingival graft harvesting [7]. However, wound healing of the palate may be delayed after harvesting, because palatal tissue undergoes secondary healing that requires the spontaneous migration of epithelial cells from the margins to the center of the wound [8]. During the healing process, patient discomfort remains a primary concern [9].Therefore, it is critical to develop a wound healing material which protects the wound site and promotes wound healing.

*Angelica gigas* Nakai (AG) is a medicinal herbal plant that belongs to the genus *Angelica* L. in the family of Umbelliferae [10] and is commonly found in Korea [11]. AG has traditionally been used in oriental medicine because of its biological benefits. A recent review showed that AG contains several chemical components, such as pyranocoumarins (decursin and decursinol algelate), other coumarins, phthalides, flavonoids, and polysaccharide, and pyranocoumarins mainly produce the biological effects of AG [12]. An in vivo study showed that topical AG treatment reduced collagen destruction and tissue necrosis factor-α and interleukin-1β expression in rats with ultraviolet-induced skin damage [13]. A previous study found that AG showed anti-inflammatory effects on lipopolysaccharide (LPS)-stimulated RAW 264.7 cells [14]. Another in vitro study showed its wound-healing effect in human keratinocytes [15], suggesting a potential contribution of AG to oral wound healing.

The selection of the extraction method is important to maintain the biological properties of medicinal plants. Traditional extraction methods include percolation, ultrasound-assisted extraction, and Soxhlet extraction [16]. Recently, advances in pharmaceutics have led to innovations in drug delivery. Hot melt extrusion (HME) is a technology that produces solid dispersions to improve the bioavailability and solubility of water-insoluble active ingredients [17]. HME technology has several advantages over the traditional extraction methods. As HME is a solvent-free process, immunogenicity or other toxic effects can be minimized [18]. Furthermore, HME is easily customized for pharmaceutical uses [19], including oral delivery [20]. Several studies have demonstrated the biological effects of AG prepared with HME technology [21,22]. A previous study demonstrated that HME increases the water solubility of active ingredients in AG and enhances biological activity [21]. Therefore, this study aimed to evaluate the effects of AG prepared from HME technology on oral wound healing in an in vitro model. The null hypothesis was that oral wound healing is not significantly influenced by AG prepared using HME technology.

## 2. Materials and Methods

### 2.1. Preparation of AG by HME Technology

**Figure 1:**
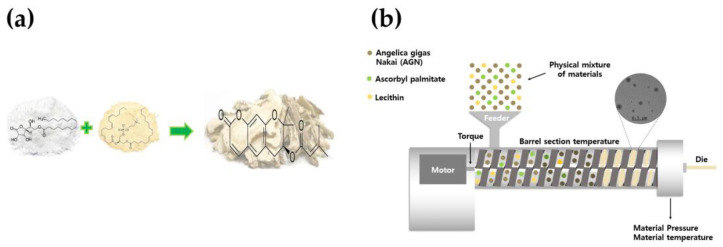
Preparation of AG by HME technology. (**a**) AG with added excipients; (**b**) a schematic design of the HME process.

AG powders were processed by the HME process, as previously described, with some modifications [21]. AG was applied to copper metal, and fully loaded into a twin-screw extruder (STS-25HS, co-rotating intermeshing twin-screw extruder, Pyeongtaek, Republic of Korea). A circular die (1 mm diameter) was mounted on the extruder. The powder was fed into the extruder at a feed rate of 150 pm and a pressure of 15 bar. The temperature profile from the feed zone to the injected dye of the extrusion mold was 100 °C. After completing the HME process, the extrudates were dried in an oven (SCOV-150, Sungchan Science, Pocheon, Republic of Korea) at 70 °C for 48 h, and the pulverized powder was stored for further experiments (Figure 1).

**Figure 2:**
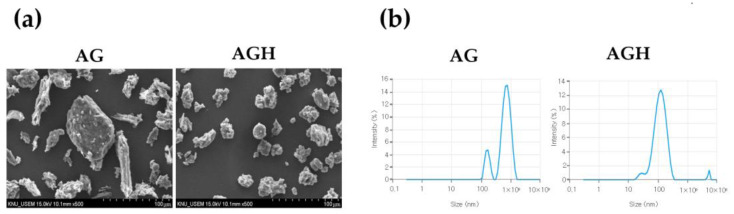
Confirmation of the particle characterization. (**a**) Scanning electron microscopy (SEM) images. SEM images at 500× magnification show the homogeneity of AGH particles. (**b**) Dynamic light scattering (DLS). The results show that AGH is more homogeneous in size distribution and smaller in size than AG.

To characterize the particles, AG and AG via hot-melt extrusion (AGH) samples were coated with platinum for 60 s using a sample-coating machine (EM ACE600, Leica, Germany) and photographed under a scanning electron microscope (SEM; S-4800, Hitachi, Japan). In addition, dynamic light scattering (DLS) was performed using a ZSP (Malvern Instruments, Malvern, UK) to determine the size distribution (Figure 2).

### 2.2. Culture of Human Gingival Fibroblasts

Human gingiva fibroblast (HGF; ATCC, PCS-201-018™) cells were purchased and incubated with Dulbecco’s Modified Eagle’s (DMEM; GibcoBRL, Life Technologies, Grand Island, NY, USA) supplemented with 10% fetal bovine serum and 1% antibiotics (100 U/mL penicillin and 100 μg/mL streptomycin) at 37 °C, 5% CO_2_. The cells were further incubated until confluency. Finally, cells from the 4th to 6th generations were used in this study.

### 2.3. Cell Viability Test

Cell viability was measured using a cell counting kit-8 assay (CCK-8; Dojindo Molecular Technologies, Rockville, MD, USA). HGF cells were seeded into 96-well plates at a density of 5 × 10^3^ cells/well and incubated at 37 °C, 5% CO_2_ for 24 h. The AG and AGH samples (*n* = 5) were treated with various concentrations for 24 h. The CCK-8 solution (20 μL) was added to the plates and incubated for 2 h at 37 °C. To compare cell viabilities, optical density at 450 nm was measured using a microreader (AMR-100; Allsheng, Hangzhou, Zhejiang, China).

### 2.4. Nitric Oxide (NO) Assay

For evaluating anti-inflammatory effects of AG and AGH, a NO assay was performed following the standard method with some modifications [23]. The cells (*n* = 4) were seeded into 6-well plates with a density of 2 × 10^5^ cells/well, incubated for 24 h, and treated with 10 μg/mL of LPS from *Porphyromonas gingivalis* (InvivoGen, San Diego, CA, USA) for 24 h. AGH solutions (125 μg/mL) were added into the wells 1 h before LPS treatment. NO production was measured using a NO Plus Detection Kit (iNtRON Biotechnology, Inc., Seoul, Republic of Korea). Then, 100 μL of the supernatants were placed in 96-well plates, followed by 50 μL of N1 buffer, and incubated for 10 min at 37 °C. Next, 50 μL of N2 buffer was added and the optical density was measured at 540 nm after 10 min using a microreader (AMR-100; Allsheng, Hangzhou, Zhejiang, China). The quantification of NO production was calculated from an 8-point nitrite standard curve according to the manufacturer’s protocol.

### 2.5. Cell Proliferation/Cell Migration Assay

For evaluating cell proliferation/cell migration, the cells were seeded on silicone inserts (SPLScar™ Block, SPL Life Sciences, Gyeonggido, Republic of Korea) in 35 mm culture dishes and incubated for 24 h at 37 °C. The silicone was then removed using sterile forceps. Then, either 125 μg/mL of AG or AGH was treated and then incubated. The gap lengths between the margins (*n* = 8) were imaged and measured with microscopy for 24 h. The open wound area (%) was calculated as the ratio of the gaps between the evaluation time and 0 h.

### 2.6. Quantitative Real-Time Polymerase Chain Reaction (qRT-PCR)

To assess the expression of biomarkers involved in oral wound healing, HGF cells were seeded in 6-well plates (5 × 10^5^ cells/well) for 24 h. The cells (*n* = 3) were then exposed to either 125 μg/mL of AG or AGH for 24 h, and RNA was extracted using easy-BLUE™. Total RNA was reverse-transcribed to cDNA using Maxime™ RT PreMix (iNtRON Biotechnology). qPCR was performed on a Step One Plus real-time PCR system (Applied Biosystems, Thermo Fisher Scientific, Inc., Waltham, MA, USA) using Power SYBR™ Green PCR Master Mix (Applied Biosystems). cDNA levels were calculated using the 2^–ΔΔCt^ method and normalized to glyceraldehyde 3-phosphate dehydrogenase (GAPDH) levels. 

The following primer sequences were used: vascular endothelial growth factor (VEGF), (F) 5′-TCACAGGTACAGGGATGAGGACAC-3′, (R) 5′-CAAAGCACAGCAATGTCCTGAAG-3′, alpha-smooth muscle actin (α-SMA), (F) 5′-GCCTGATGGGCAAGTGAT-3′, (R) 5′-TGCTGGAAGGTGGACA-3′, alpha 1 type I collagen (COL1α1), (F) 5′-CCCGGGTTTCAGAGACAACTTC-3′, and (R) 5′-TCCACATGCTTTATTCCAGCAATC-3′.

### 2.7. Statistical Analysis

IBM SPSS Statistics (version 20.0; IBM Corp., Armonk, NY, USA) was used to analyze the data. The Shapiro–Wilk test was performed to check the normality of the data. A one-way analysis of variance (ANOVA) was used to determine significance, followed by a post hoc analysis using the Tukey test. *p*-values < 0.05 were considered statistically significant.

## 3. Results

**Figure 3:**
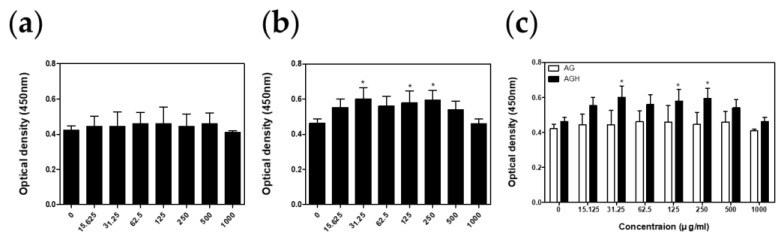
Cell viability tests. (**a**) Cell viability test of AG (*n* = 5). (**b**) Cell viability test of AGH (*n* = 5). The cell viabilities were compared to that in the control (* *p* < 0.05 vs. control). (**c**) Comparison of cell viabilities between AG and AGH (* *p* < 0.05 vs. AG).

**Figure 4:**
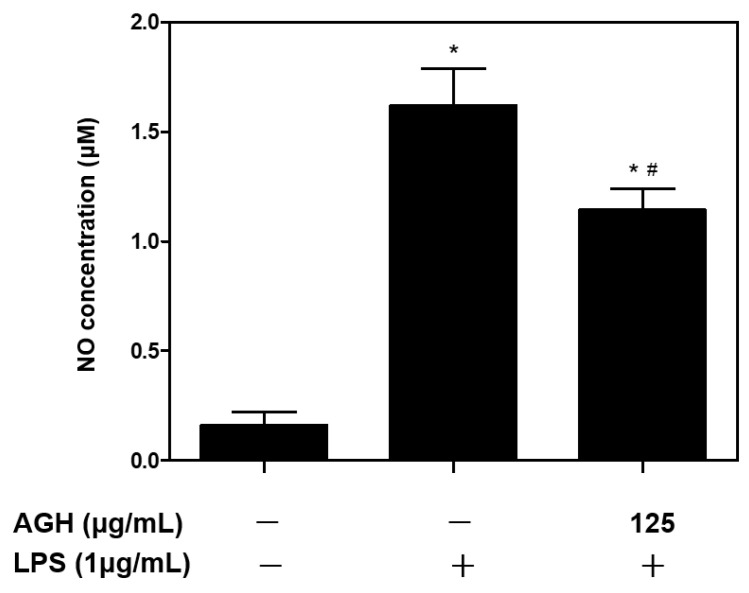
NO production (*n* = 4). AGH significantly decreased NO production from LPS stimulation. * *p* < 0.05 vs. control, ^#^ *p* < 0.05 vs. control + LPS.

**Figure 5:**
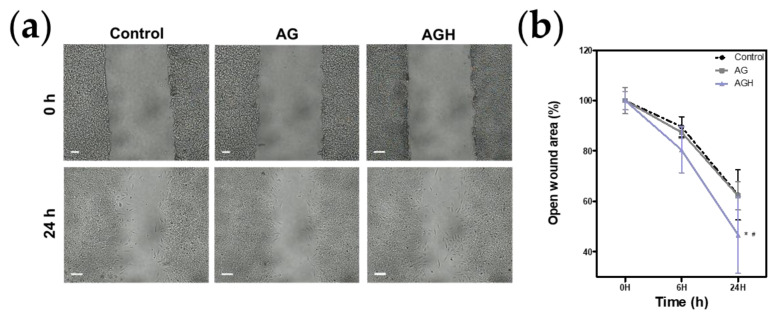
Cell proliferation/cell migration assay (*n* = 8). (**a**) Microscopic view. (**b**) Analysis of open wound area (%). The open wound area (%) was calculated as the ratio of the gaps between the evaluation time and 0 h in each group. The results show that AGH significantly decreased in the open wound area (* *p* < 0.05 vs. control, ^#^ *p* < 0.05 vs. AG).

**Figure 6:**
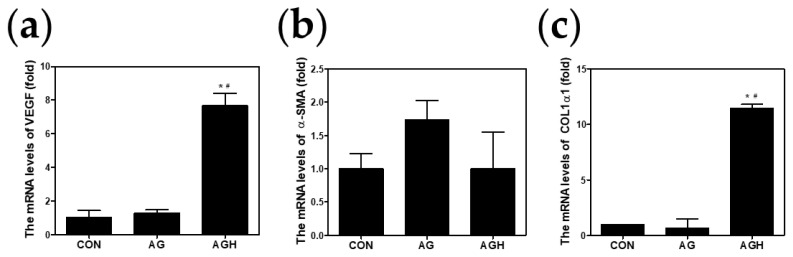
Expression of wound healing-related biomarkers (*n* = 3). (**a**) VEGF. (**b**) α-SMA. (**c**) COL1α1. The results show that AGH significantly upregulates the VEGF (*p* = 0.002 vs. control, *p* = 0.002 vs. AG) and COL1α1 (*p* = 0.001 vs. control, *p* = 0.001 vs. AG) expression levels (* *p* < 0.05 vs. control, ^#^ *p* < 0.05 vs. AG). CON, control (no treatment); AG, *Angelica gigas* Nakai; AGH, *Angelica gigas* Nakai from hot-melt extrusion technology.

Cell viability tests revealed that AGH had no cytotoxic effect on human gingival fibroblasts (Figure 3). The results from the NO assay showed that AGH significantly decreased NO production under LPS stimulation (Figure 4). The cell proliferation/migration assay showed that AGH significantly decreased the open wound area compared to that in the control after 24 h (*p* = 0.022). However, there were no differences in the open wound areas between the AG and control groups (Figure 5). Figure 6 shows the expression of the biomarkers associated with oral wound healing. We observed a significant upregulation of both VEGF (*p* = 0.002) and COL1α1 (*p* = 0.001) in AGH compared to that in the control and AG. However, there were no significant differences in α-SMA expression among the materials.

## 4. Discussion

This study evaluated the wound healing effects of AGH in vitro. AGH significantly decreased the open wound area and upregulated the expression of biomarkers involved in oral wound healing. Thus, the null hypothesis was rejected.

In this study, HME technology was used. Recently, HME has been considered a convenient processing technology for use in various industries such as pharmaceuticals, polymer science, and food science [24]. HME has the potential to enhance the water solubility and oral bioavailability of the active molecule [25]. Moreover, HME allows for the attainment of distinct particle characteristics, including the uniform distribution of processed extrudates and consistency in particle size [26]. HME is a sustainable processing system that decreases organic solvent usage, minimizes the risk of toxicity, and provides advantages of a single and continuous process [27]. A previous study regarding HME-orientated antihypertensive molecules revealed high levels of bioavailability in a rat model [28]. Another study on HME-orientated resveratrol confirmed an improvement in the solubility and oral bioavailability of resveratrol [29]. A previous animal study also confirmed improvements in the oral bioavailability and biological effects of cyclosporine through HME [30]. Together with these findings, utilizing HME technology for drug delivery in oral environment will be beneficial.

In this study, AGH treatment significantly reduced LPS-induced NO production. NO is a major signaling molecule that participates in the inflammation process [31]. Previous evidence has suggested that NO production is positively related to pro-inflammatory cytokine expression [32,33]. Based on these findings, it may be assumed that AGH improves oral wound healing by inhibiting inflammation. Inflammation is fundamental for oral wound healing in the early phase of oral wound healing because it brings macrophages and neutrophils to the wound site and thereby induces the release of cytokines associated with wound healing [34]. However, prolonged inflammation inhibits wound healing by producing toxic free radicals and reactive oxygen species, as well as increasing the risk of infection [4]. 

After the inflammation subsides, the oral wound healing process progresses through two phases: proliferation and remodeling. During the proliferation stage, various types of cells, including endothelial cells, fibroblasts, and epithelial cells, migrate to the wound site and induce angiogenesis and re-epithelialization [1]. Briefly, fibroblasts produce granulation tissue, with highly vascularized, endothelial cells building structural support for the wound, and epithelial cells induce re-epithelialization, in turn [35]. Thus, cell migration is fundamental in the process of oral wound healing.

In this study, the cell migration assay confirmed that AGH accelerated HGF migration; however, AG did not accelerate HGF migration compared to the control. This finding indicates that AGH may produce a favorable environment for facilitating oral wound healing. The sustained release of the biological effect of AG through HME technology could be a possible explanation. HME technology aims to facilitate targeted drug delivery. Recent studies have demonstrated that the HME technology is a feasible vehicle for effective drug release [21,36].

In this study, AGH upregulated the expression of biomarkers associated with oral wound healing. VEGF expression was significantly higher in AGH. Considering that VEGF is a key factor in angiogenesis during the proliferation stage [5], this finding indicated that drug delivery by AGH was steadier and more effective. VEGF is associated with angiogenesis, epithelialization, and collagen synthesis [37]. Similarly, COL1α1 expression was also highlighted in AGH, which was correlated with the results of previous studies which reported that AG increases type I collagen synthesis in human dermal fibroblasts [38]. This finding indicates that AGH may act as a collagen synthesis stimulator and facilitate oral tissue regeneration.

Our results showed that AGH may accelerate the oral wound healing process. However, this study has some limitations. First, the biological effects of AGH during the inflammatory phase were not determined. The precise mechanism by which AGH affects wound healing has not yet been fully elucidated. Further evaluation of the protein levels is needed.

## 5. Conclusions

In conclusion, oral wound healing effects of AGH were assessed in this study. Within the limits of this study, AGH accelerated the oral wound healing process and showed significantly upregulated expressions of VEGF and COL1α1. This finding suggests that AG delivery via HEM technology holds benefits.

## Data Availability

All data analyzed during this study are included in this published article.
